# Parents’ and healthcare professionals’ perceptions of the use of live video recording in neonatal units: a focus group study

**DOI:** 10.1186/s12887-020-02041-9

**Published:** 2020-04-01

**Authors:** Aude Le Bris, Nadia Mazille-Orfanos, Pauline Simonot, Maude Luherne, Cyril Flamant, Geraldine Gascoin, Gearóid ÓLaighin, Richard Harte, Patrick Pladys

**Affiliations:** 1 Department of Neonatology, University Hospital of Rennes, 35000 Rennes, France; 2 Department of Neonatology, University Hospital of Caen, Caen, France; 3 Research and Innovation Department, Paediatric Department, University Hospital of Rennes and GCS HUGO, Rennes, France; 4 Department of Neonatology, University Hospital of Nantes, Nantes, France; 5 Department of Neonatology, University Hospital of Angers, Angers, France; 6 CURAM, Human Movement Laboratory, NUI Galway, Galway, Ireland

**Keywords:** Healthcare professionals, Focus groups, Parents, Perceptions, Video

## Abstract

**Background:**

The emerging use of video in neonatology units raises ethical and practical questions. This study aims to gain a better understanding of the suitability, limitations and constraints concerning the use of live video as a tool in neonatal clinical practice. The perceptions of parents and healthcare professionals in regard to live video were examined.

**Methods:**

Nine focus groups were conducted in four neonatal units involving 20 healthcare professionals and 19 parents. Data were triangulated using transcripts and field notes and analyzed using inductive and semantic thematic analysis.

**Results:**

The seven major themes that emerged from the healthcare professionals focus groups were (i) the impact of video recording on healthcare professionals’ behavior; (ii) the impact on parents; (iii) forensic issues;(iv) guarantee of use; (v) benefits for the newborn; (vi) methodology of use; and (vii) technical considerations & feasibility. The five major themes that emerged from parents focus groups were (i) benefits for the newborn and care enhancement; (ii) impact on parents and potential benefits in case of newborn child/parent separation; (iii) informed consent and guarantee of use;(iv) concern about a possible disruptive impact on healthcare professionals; and (v) data protection.

**Conclusion:**

Both parents and healthcare professionals found video recording useful and acceptable if measures were taken to protect the data and mitigate any negative impacts on healthcare professionals.

## Background

Technology is increasingly present in hospitals [1, 2], with the emergence of electronic medical records and e-prescriptions [3], the use of telemedicine [4] and the use of local networks to share medical data. Video recording has also shown its potential in the field of medical training through simulation [5] and e-learning [6] and has started to emerge in healthcare services. While the use of video is currently not common practice in neonatology, it has increased to meet new needs such as photoplethysmography, video laryngoscopy or webcams to enable virtual visit [7–9]. Webcams have been used [10–14] for several years either to limit the impact of parent-child separation or when parental presence is limited to promote early emotional bonding and reduce separation anxiety.

Few data are available on how parents and healthcare professionals perceive this technology. Yeo et al. [11] showed through the use of surveys that this technology is easily accepted and enthusiastically adopted by parents. Cameras placed in the newborn’s bed were activated when the newborn was not receiving active care, and images were accessible in real-time by parents. Hawkes et al. [12] surveyed parents and healthcare professionals before implementing a webcam monitoring system. Most parents were in favor of this implementation, in contrast to healthcare professionals who were mostly unfavorable. Healthcare professionals were concerned by their lack of familiarity with such a system, the risk of privacy breach, and the potential stress created due to the presence of the webcam. Kerr et al. [13] found that parents and healthcare professionals consider parents’ direct access to a recording of their newborn child an important improvement in neonatology.

Video recording is not standard of care in our unit but has been used in a few research projects notably as part of the Horizon 2020 Digi-NewB project [15]. Its aim is to improve care for newborns through the development of a next generation video and sound monitoring system. In this project the camera is in the newborn’s incubator. The implementation of this research project has raised ethical questions about the widespread use of video in the daily care of newborns. The few studies that are available in this field are based on questionnaires or individual interviews. Therefore, in order to continue and expand the use of video in the neonatal intensive care unit we aimed to further our understanding of parents’ and healthcare professionals’ perception of video recording through the use of focus groups. We conducted this study to explore the issues associated with the use of video recording in clinical practice in neonatology.

The main objective of this study was to analyse how parents and healthcare professionals perceive the use of video recording in neonatology units, in order to improve our knowledge of its potential impact in terms of human perception, benefits, limitations and constraints.

## Methods

### Design

We performed a multicentre qualitative focus group study to collect feedback on the use of video recording in neonatal clinical practice, following COREQ guidelines [16]. We chose this approach to generate data on the collective perception as well as the personal opinions and experiences of each participant.

We included in the study parents of newborn who had been hospitalized in the unit for at least 2 weeks and still hospitalized at the time of the interview. The healthcare professionals included all worked in one of a neonatology unit involved in the study including the neonatal unit of the moderator for four of them. The focus groups included physicians, nurses, health managers and psychologists.

Focus groups with healthcare professionals were conducted in one centre in Ireland (Galway) and two centres in France (Angers and Rennes), while focus groups with parents of newborns were conducted in three centres (Angers, Nantes and Rennes) in France. This study took place between March 2018 and May 2018.

We chose two distinct focus group categories (healthcare professionals and parents) to facilitate open discussions and obtain different perspectives. Each professional focus group included different healthcare professions (physicians, nurses, psychologist, health manager) to obtain different experiences and characteristics to enable the collection of a vast array of perceptions from the participants.

### Ethical considerations

The study was approved by the Rennes University Hospital Ethics Committee (reference number 18.21) and all participants gave their informed consent in writing before participating. A physician was responsible for explaining the research project to potential participants. The same physician also had to submit an email newsletter on the purpose and conduct of the research. Any individuals who expressed an interest in participating in the focus groups and who met the inclusion criteria were included. Participation was on a voluntary basis and participants were informed that they could withdraw their consent to participate or their feedback at any time. All interviews took place within the hospital in a private room and in accordance with the principle of confidentiality.

### Data collection

Each focus group session was facilitated by a moderator with experience in conducting focus groups and familiar with the subject of the study. The session was semi-structured in nature, with a pre-defined list of open-ended questions being asked to the participants during each session (Table 1**)**. To ensure trustworthiness we used the same focus group discussion guide in every session, this guide was piloted before the start of the study. The sessions were audio recorded upon receiving the consent of each participant. The audio recordings were then transcribed verbatim and deidentified.

**Table 1:** Topics covered in focus group with parents and health professionals

*- What does the use of video mean to you in your daily life, what is its use?*	
*- What would be its contributions to hospital services, particularly in neonatology?*	
*- What are the obstacles to its use for you?*	
*- Does the use of video seem acceptable to you and your personal conviction?*	
*- Would you like to have access to this video? In what condition, for what purpose?*	
*- Would there be an impact on your behaviour?*	

Throughout the session, the moderator summarised and reformulated the results and presented them back to the participants to ensure the information was accurate and that their points had been understood correctly. This step was required to ensure the accuracy of the subsequent analyses. At the end of the session, participants completed a short quantitative questionnaire in order to capture their socio-demographic characteristics. The main moderator in Galway was an English-speaking researcher from NUI Galway, while in France the main moderator was a French researcher. The moderator from France also co-moderated the session in Galway. Observers were present to take notes at each session.

Internal validation of the data was carried out by the moderator, the observer (who also transcribed the audio recording verbatim), as well as by a neonatologist who also coded the interviews. We collected and analysed data iteratively. Data collection continued until saturation was achieved, i.e. no new themes were occurring in either staff [17].

### Data analysis

Data were analysed using an inductive approach to identify patterns that emerged from the data. Three members of the research team (AL, PS, NM) independently read the transcripts and generated codes. The coding unit were full sentences, mostly because interviews were conducted both in French and English preventing a word by word translation. Codes were reviewed and revised by the investigators. Codes were then sorted into themes. At each step, the investigators met to assess similarities and differences in analyses until a consensus was reached on all the themes. A list of themes and sub-themes was then generated and extracted in tabular form. Constant comparative analysis was used to assess overall saturation [18]. The authors collectively selected and presented verbatim quotes to illustrate the thematic findings in tabular form. For the French verbatims quotes, we carried out a double translation to ensure its correct meaning.

After the inductive coding was completed, and themes were established, we used a qualitative summative content analysis [19] to determine the most prominent themes.

We coded the data from transcripts using the Saldaňa methods [20] and evaluated the frequency of each theme using the qualitative data management software NVivo® 12 Plus (QSR International).

To ensure trustworthiness of the coding and analysis of the data, findings were discussed among authors. Transferability, described as the ability to apply findings to similar contexts, was addressed through a clear description of the participants’ characteristics, settings and research process.

## Results

### Participants’ characteristics

#### Parents

A total of nineteen parents participated in the study. Five focus groups were conducted, with each focus group consisting of 4 participants, except in one case where one parent became unexpectedly unavailable. The sessions lasted 32 to 40 min. The profile and the characteristics of the participating parents and a description of newborns’ diagnosis are presented in Table 2. None of the parents had prior experience with hospitalisation in neonatology.

**Table 2:** Characteristics of parents (*n* = 19)

**Parental Role**	Mothers	15
	Fathers	4
**Age (years)**	20–30	7
	30–40	12
**Educational background**	Primary education	1
	Secondary education	6
	Higher education	12
**Marital status**	Married / living with partner	19
	Single	0
**Use social networks**	Yes	17
	No	2
**Experience of video at work**	Yes	6
	No	12
	Not specified	1
**Use of personal video**	Yes	19
	No	0
**Experience of hospitalisation with another child**	Yes	0
	No	19
**Diagnosis of newborn hospitalised**	Prematurity	14
	Malformative pathology	5
**Use of video in research project**	Yes	7
	No	12

#### Healthcare professionals

Twenty healthcare professionals participated in the study. Four focus groups were conducted with each focus group consisting of 4 to 6 participants. The duration of the interviews ranged from 36 to 62 min. The characteristics of the healthcare professionals are summarised in Table 3.

**Table 3:** Characteristics of health professionals (*n* = 20)

	Gender (F/M)	Average (years)	Average work experience (years)	Video experience in hospital	Private use of social networks	Private use of the video
**Staff (*n*** **= 20)**	17/3	42 (20;56)	16 (1;31)	13	10	18
**Nurses (*n*** **= 11)**	11/0	42 (20;56)	18 (1;31)	7	6	9
**Doctors (*n*** **= 8)**	5/3	41 (27;52)	11 (2;25)	6	4	8
**Psychologist (*n*** **= 1)**	1/0	39	14	0	0	0

### Thematic analysis

We obtained a saturation of the data, i.e. all the themes were found in each group, and we did not find a new theme after several analyses of the data.

The themes extracted from the data, classified in order of frequency, are presented in Table 4. Five main themes arose from the analysis of the data from the parents’ focus groups. Seven main themes were identified in the healthcare professionals’ groups. Four themes were found to be common between both groups.

**Table 4:** Themes presented by frequency of occurrence

Parents	Healthcare professionals
1.Best interests of the child and improved care	1.Concern for the possible impact on caregivers
2.Impact of images on parents	2.Impact of images on parents
3.Informed consent and guarantee of use	3.Forensic dimension
4.Concern for the possible impact on caregivers	4.Inform consent and guarantee of use
5.Data protection and privacy	5.Best interest of the child and improved care
	6.Ways of use: practice improvement, teaching, research
	7.Technical aspect and feasibility

Quotes illustrating each theme are presented in Table 5.

**Table 5:** Example quotes for each theme

Themes	Focus group
**Best interests of the child and improved care**	
“It should really always be used in an effort to improve care [...]”, “It should always be in the patient’s interest, I think.”	Parent, Nantes
“We have children who leave quickly [...] if we have the means to spot this upstream, yes, clearly there is a real benefit, it’s worth it...”	Professional, Rennes
**Impact of images on parents**	
“I see with my wife; I took several video clips [...] she watched them a lot of times so it’s true that it can create a bond.”	Parent, Rennes
“It also seems a little anxious to me, actually, we’re not professionals [...] we can see things that worry us when in fact it’s not worrying.”	Parent, Rennes
“There could be a drift [...], to be watching all the time and then when you’re at home, you should also cut, recharge...”.	Parent, Rennes
**Concern for the possible impact on healthcare professionals**	
“I put myself in their shoes, maybe I’d feel a little pressure, a little eye above my head to see if I’m doing my job well.”	Parent, Angers
“If baby is sleeping, we don’t go there... but this can be an opportunity to have a discussion with the mother.”	Professional, Rennes
“Then we finally forget that the video is there”.	Professional, Galway
**Informed consent and guarantee of use**	
“In fact, it is rather up to them (the professional) to give their agreement or not”	Parent, Angers
“there is a need to know where the limits are”	Professional, Rennes
**Data protection and privacy**	
“You shouldn’t be able to access it anywhere, anyhow either.”	Parent, Rennes
**Forensic dimension**	
“[...] during a trial for a death, can there not at some point be a lifting of secrecy? A lawyer may be able to negotiate successfully to access the images”	Professional, Galway
“That’s what scares (me) about video recording, its possible (erroneous) interpretation.”	Professional, Angers
**Potentials use: practice improvement, teaching, research**	
“For oral problems the video would be useful for filming the feeding, see the breathing-deflutition synchronization”	Professional, Angers
**Technical aspect and feasibility**	
“if you just had to turn it on, like attaching a sensor. I think it would work.”	Professional, Rennes

#### Four themes common to both groups

- *Best interest of the child and improved care*

Naturally, the child’s best interest was of prime importance to parents. The introduction of video was seen as a potential mean to improve the child’s care through improved understanding of the child’s behaviour and a better assessment of the child’s need for personalized care. The use of video could contribute to and improve already available monitoring tools such as the patient monitoring scope, or the NidCap. According to parents, if the camera offers an advantage for healthcare professionals and hence improves the care of the child, then it is an acceptable addition.

While the interest of the child was not the first issue raised by healthcare professionals, it was a main concern, with some ethical questions about what is best for the child. The benefits of the technology in terms of optimising the care of the newborn, either diagnostically or by enabling the personalization of care through a better understanding of the newborn’s behaviour, was an important discussion among healthcare professionals.

- *Impact of images on parents*

Parents responded mostly positively to the use of video as a webcam to view images of their child. The reasons provided were: the facilitation of parent-child bonding in situations of forced separation (reduced mobility due to a C-section, mother-child hospitalization in two different centres), the feeling of being close to their baby, parental reassurance by monitoring the well-being of their child at all time and finally a better grasp of a highly technical environment around the newborn. Concerns were also raised in both groups. For example, in situations where access to live video is not available, parents could potentially worry that a serious event has occurred creating a source of additional stress. Parents indicated that it would probably be necessary for the images to be explained by the healthcare professionals, referred to as “experts”. The contextualization of images by professionals was a guarantee requested by parents. One example cited by the parents was if their child was in distress, requiring an intubation or similar procedure, the “shocking” images without appropriate explanations could lead to stress.

Parents also expressed the fear of hypervigilance if continuous home connection was available, with possible fatigue. Some parents were worried that they would no longer be able to benefit from “real” rest time outside the room, which would have a significant physical and psychological burden.

The issue of privacy was also widely raised. The camera recordings were seen as potentially intrusive. Of particular concern was the effect of the potential intrusion on intimate moments between parents and child, such as skin-to-skin moments or during breast-feeding. The camera was then viewed as a ‘third-eye’. In addition, parents were worried about the confidentiality of their own conversations around the system.

- *Concern for the possible impact on healthcare professionals*

The potential impact of the system on healthcare professionals was a concern for parents. They feared that healthcare professionals would feel increased anxiety while carrying out care under video surveillance, thereby increasing the risk of medical error.

The presence of the video was also seen as potentially harming the parent-healthcare professionals’ relationship by reducing the amount of time professionals spend in the room.

Access to live video was also seen as an opportunity to optimize how healthcare professionals target interventions with respect to sleep phases, thereby reducing unnecessary noise and light exposure to the child. But this lesser presence could also be detrimental with less time spent interacting with parents, as professionals will often combine a visit to the baby with a chance to discuss care with parents. This time of exchange was considered by the parents as privileged time with an expert who reassured them, but also allowed for the maintenance of social bonds which are often fragile during the period of hospitalization.

According to parents, having healthcare professionals under constant webcam surveillance*,* could lead to a loss of trust between parents and professionals.

This theme was the primary concern of healthcare professionals. A possible change in behaviour of the healthcare professionals could occur with the presence of the camera by fear of “doing something wrong” or “being judged”, even if this issue was mitigated by the fact that the professionals were already used to caring for newborn in front of parents. The other concern raised was the risk of self-censorship when interacting with the newborn because of the unpleasant feeling of being ‘heard’, with the potential loss of more genuine humane interactions such as singing a lullaby or adopting a more familiar attitude towards the child. However, positive aspects were also identified, such as a more rigorous approach to hygiene and the potential for personalized and behavioural care to be provided to newborns. The impact on the healthcare professionals’s time in the child’s room was another topic of discussion. However, all professionals agreed that there is likely an “adaptation” phase to video recording, which seemed to be confirmed by professionals who are already using video in their clinical practice.

- *Informed consent and guarantee of use*

Both healthcare professionals and parents mentioned that information and consent to video recording were essential prerequisites. They emphasized the need to obtain consent from both parents and exposed healthcare professionals.

Both parties shared the same concerns about the requirement to provide the purpose of the recording and the guarantee of use, with the two main questions being: who will have access to the videos and why.

The child’s consent was an issue raised only by healthcare professionals. In this situation, healthcare professionals wondered who the guardian of the child’s best interest would be.

#### One theme exclusively mentioned by parents

- *Data protection and privacy*

Data protection was the last point raised by parents with the fear of data being compromised when WIFI and external network are use. This is an issue that is widely discussed but not well thought through from a technical and feasibility perspective.

#### Three themes only mentioned by healthcare professionals

- *Forensic dimension*

The forensic aspect was widely discussed by the various professions within the group of participating healthcare professionals (psychologist, doctors, nurses). By analysing the themes per profession, the legal dimension is the first theme mentioned by doctors, fearing a possible legal course of action by parents. This was also widely addressed by the other professionals interviewed, but in the instance of doctors, there was a particular fear of retaliation from the institutional hierarchy. According to the healthcare professionals, video could facilitate this course of action because of the “evidence” images can provide. There was a concern that a third party (typically lawyers) would erroneously analyse images taken out of their context in the event of adverse medical events. Another fear was the forensic impact due to the unavailability of images during technical problems as this could be interpreted as a desire on the part of the healthcare professionals or other actors to “hide” some events.

- *Potential use: practice improvement, teaching, research*

The suggestions of potential ways to use video recording varied depending on each interviewee’s occupation and experience. Nurses mentioned how complementary videos are with the tools already available, one example being the combination of video with NidCap observations in order to refine evaluations of oral quality or respiratory maturity. Doctors also spoke of the video as a complementary diagnostic tool to cardiorespiratory monitoring. However, in all professional categories, the interest in simulation and e-learning teaching to improve clinical practices was high.

- *Technical aspect and feasibility*

One technical concern with the introduction of video recording was equipment maintenance and training of the staff in charge of this tool. Professionals mentioned the need for assistance from biomedical engineers and the designation of a charge person for the management of this technology. More practical and performance related questions were also addressed, such as the focus of the camera or the different modes.

## Discussion

The purpose of this study was to explore the different perceptions of parents and healthcare professionals regarding the use of video in neonatal units. Although the two groups of participants have different point of views, all consider video to be useful and acceptable under certain prerequisites, namely the assurance of informed consent, robust data protection and to limit potential negative impacts on healthcare professionals.

### Negative outcomes of video

The first concern raised by both groups of participants was the effect that the video would have on healthcare professionals. Parents would be reluctant to use video if it had a negative impact on their child’s care, and if it had the potential to negatively modify the behaviour of healthcare professionals. The findings in this study align with the findings from an American study [14] on the impact of the use of webcams in neonatology on nurses’ workload. Of specific concerns were the increase in stress, material handling time and time spent on the phone with parents to assist them in interpreting the images. This team provided training for healthcare professionals before this technology could be widely used, particularly on how to use and maintain the equipment.

The question of intimacy, raised by parents, was also an important topic highlighted in the study of L. van Lonkhuijzen et al. [21] where video was used in the birth room. The proposed solution was to focus the camera’s frame on the new-born child and study only him, which is also a solution strived for within the Digi-NewB project. Thus, skin-to-skin or breastfeeding moments would not be captured by the camera. They also proposed that audio recording could be interrupted at the parent request.

Another common concern is the impact that images could have on parents in the case of on-going remote access to live video in acute situations (resuscitation, technical procedures). This could be prevented with the planned and anticipated shutdown of the cameras during any emergency care procedure [14] or with an automatic display of a message on the screen indicating that a procedure is in progress when the video is turned off [13].

In addition, the obligation to give consent, to provide the conditions of use, the purpose of the tool and access to the video, was already required in a Dutch study [21]. Data protection is also essential at a time when hacking is frequent [22]. As a result, close collaboration between the IT department and the staff is essential [12]. The use of a secure portal with a unique secure login and password for each newborn is an option [11].

Moreover, only the healthcare professionals spoke about the forensic issue. They fear that the images will be used for legal purposes if adverse medical events occur. This point is widely discussed in an Australian review [23] where doctors and nurses are concerned that video recording could provide evidence in case of medical or paramedical errors. The main source of disputes is to ensure that nothing is hidden, and video therefore reduces this risk as the information becomes then available. Video recordings could be used to provide evidence of good practice rather than to track possible errors [21]. O’Donnel et al. [24] suggested to make the acquired images anonymous. They propose to depersonalize the registration as much as possible during storage (no name, no date, no place), to focus the camera solely on the baby and the professional’ forearms and introduce a specific legislative framework.

### Positive outcomes of video

The positive perception of video recording by both parents and healthcare professionals is in agreement with other studies [11, 13].

Several elements justify why the potential introduction of such a system was well received. Better care for the child through a more refined interpretation of his or her behaviour seems to be an important element. Indeed, it could allow early detection of particular events as well as individualisation of care in sync with the newborn’s abilities. Other positive points include the use of webcam mode, which would promote early parent-child bonding [11, 25] and allow parents to better understand their baby’s behaviours. Webcam use is seen as a good palliative tool in situations of forced separation [13, 26]. Kerr et al. [13] evaluated how parents responded to webcam use. They described an increased sense of proximity and responsiveness to their child, emotional well-being, improved physical recovery and the opportunity to introduce the child to family and friends.

In our study, as in the literature, parents also consider video as a tool to better understand the technical environment around their baby. This has a positive effect on stress induced by all the equipment needed for care and supervision [27]. Thus, webcam use seems to be an acceptable use when there is a process of early separation between parents and child that could have a long-term impact on the relationship.

Another significant advantage raised by both groups is the reduction of unnecessary professionals’ interventions in the child’s room. These interventions are typically sources of environmental pollution (noise, light) with a proven impact on the neurodevelopment of premature infants [28]. This is seen as an improvement in the care of the new-born. Finally, video is described as a complementary monitoring tool such as the patient monitor scope, NidCap observations or EEG-video.

### Strengths and limitations

Focus group data collection allowed the comparison of a large number of opinions. This comparison was made possible by conducting separate focus groups for parents and healthcare professionals exposing them to the same questions.

Studies on this subject are rare, making the focus and approach of this study original. Especially the parents’ perceptions which is not or rarely studied. It is hoped that this study will contribute to the small but growing body of literature which already exists on the subject and inform future implementation of camera systems in critical care units. Strategies used to ensure trustworthiness of analysis included triangulation in data collection and multiple coders engaging in regular peer debriefing. Diversity of roles and perspectives within the research team, ensured inter-rater reliability.

However, the results of this study must be taken with the following considerations. Inclusion of subjects was done on a voluntary basis introducing the risk of recruiting participants with certain characteristics or participants with strong positive or negative views regarding video recording. Some participants had already been exposed to video recording as part of a previous research project. The study design was implemented to include in each focus groups participants with different characteristics including participants with or without previous exposure to video recording. However, this difference in experience could have an impact on the interpretation of our results. All the parents participating in the study still had their children hospitalized in the unit at the time of the interviews, therefore it could be argued that their opinions were compromised by the emotional impact of the current situation they were in. Moreover, they all had the characteristic of being users of social networks, thereby probably more familiar with the ubiquitous use of cameras. Finally, most of the parents belonged to a high socio-professional category. These characteristics may have spontaneously made them more favourable to video recording. Unlike parents, professionals seemed less familiar with social networks. There was no mixing between the centres, so the professionals were colleagues, which may have limited their freedom of speech. The participants rarely commented on their perception of the sound from the video and focused more on images. This might be because our interview guide was more focused on the impact of video recording as a whole without specific questions regarding the impact of sound. It would be interesting to conduct a new, more specific qualitative analysis focusing on the perception of sound in the units. These factors may limit the transferability of our results.

## Conclusion

From the current study, parents and healthcare professionals seem to accept the use of video in neonatal care in a generally positive way in particular for the improvement of newborn care, but with the condition that its use is well supervised to avoid any negative impact on healthcare professionals’ behaviour or medico-legal drift.

Using the above-mentioned literature, we have proposed some possible way to improve information and acceptability (See Table 6.)

**Table 6:** Suggested elements to improve the acceptability of video

▪ Specific training of staff with video equipment, their maintenance and functionality	
▪ Focus the camera’s frame on new-born,	
▪ Allow parents to interrupt recording for privacy purpose	
▪ Stop recording during new-born care or technical procedures, but inform parents with an automatic display on the screen	
▪ Optimal data protection via a secure portal, login and password	
▪ Depersonalize recordings to the extent possible	
▪ Establish a specific legislative framework for these recording	
▪ Define in advance the duration for data storage.	

## Data Availability

The data supporting the findings are contained within the manuscript. The datasets used and analysed during the current study are available from the corresponding author on reasonable request.

## Abbreviations

NIDCAP: Neonatal Individualized Developmental Care and Assessment Program
COREQ: Consolidated criteria for Reporting Qualitative research.
